# Molecular detection of *Dirofilaria immitis, Dirofilaria repens* and *Setaria tundra* in mosquitoes from Germany

**DOI:** 10.1186/1756-3305-7-30

**Published:** 2014-01-16

**Authors:** Mandy Kronefeld, Helge Kampen, Reinhold Sassnau, Doreen Werner

**Affiliations:** 1 Friedrich-Loeffler-Institute, Federal Research Institute for Animal Health, Greifswald–Insel Riems, Germany; 2 Small Animal Practice, Berlin, Germany; 3 Leibniz-Centre for Agricultural Landscape Research, Muencheberg, Germany

**Keywords:** *Dirofilaria immitis*, *Dirofilaria repens*, Dirofilariosis, *Setaria tundra*, Setariosis, Germany, Monitoring, Mosquito, Vector, Zoonosis

## Abstract

**Background:**

As a result of globalization and climate change, *Dirofilaria immitis* and *Dirofilaria repens*, the causative agents of dirofilariosis in Europe, continue to spread from endemic areas in the Mediterranean to northern and northeastern regions of Europe where autochthonous cases of dirofilarial infections have increasingly been observed in dogs and humans. Whilst *D. repens* was recently reported from mosquitoes in putatively non-endemic areas, *D. immitis* has never been demonstrated in mosquitoes from Europe outside the Mediterranean.

**Methods:**

From 2011 to 2013, mosquitoes collected within the framework of a German national mosquito monitoring programme were screened for filarial nematodes using a newly designed filarioid-specific real-time PCR assay. Positive samples were further processed by conventional PCR amplification of the cytochrome c oxidase subunit I (COI) gene, amplicons were sequenced and sequences blasted against GenBank.

**Results:**

Approximately 17,000 female mosquitoes were subjected to filarial screening. Out of 955 pools examined, nine tested positive for filariae. Two of the COI sequences indicated *D. immitis*, one *D. repens* and four *Setaria tundra*. Two sequences could not be assigned to a known species due to a lack of similar GenBank entries. Whilst *D. immitis* and the unknown parasites were detected in *Culex pipiens/torrentium*, *D. repens* was found in a single *Anopheles daciae* and all *S. tundra* were demonstrated in *Aedes vexans*. All positive mosquitoes were collected between mid-June and early September.

**Conclusion:**

The finding of dirofilariae in German mosquitoes implies the possibility of a local natural transmission cycle. While the routes of introduction to Germany and the origin of the filariae cannot be determined retrospectively, potential culicid vectors and reservoir hosts must prospectively be identified and awareness among physicians, veterinarians and public health personnel be created. The health impact of *S. tundra* on the indigenous cervid fauna needs further investigation.

## Background

The dirofilarial species *D. immitis* and *D. repens* are the causative agents of cardiopulmonary and subcutaneous dirofilariosis, respectively, in canines, felines and other carnivores in Europe [1]. In occasional infections of humans, the nematodes may evoke subcutaneous, subconjunctival and cardiovascular lesions with infections of the lung and pulmonary blood vessels. Intra- and retroocular infections as well as infections of deeper locations such as the peritoneal cavity, the omentum and the male sexual organs may occur [1,2]. Also, rare cases of meningoencephalitis have been described [3]. Both worms are endemic in southern Europe where numbers of notified human cases of dirofilariasis have substantially increased recently [4,5]. Contrary to previous assumptions, the majority of these had probably been caused by *D. repens*[6]. In addition, an ongoing north- and eastward spread of both species has been observed, attributed to increased travel and movement of infected animals, the expansion of vector-competent mosquito species, global warming and a change in human activities [7,8]. Thus, autochthonous cases of *D. repens* infection in dogs were reported from Germany in 2004 [9] and from the Netherlands in 2008 [10], while several human autochthonous cases have been diagnosed in Poland since 2007 [11]. In 2007 and 2012, *D. repens* was again diagnosed in dogs in the German federal states of Baden-Wurttemberg and Brandenburg [12-14], suggesting that endemic circulation takes place. Moreover, autochthonous cases of *D. immitis* infection in dogs were reported from Hungary in 2009 [15], Slovakia in 2010 [16] and Poland in 2012 [17].

The primarily boreal filarial species *S. tundra* lives in the abdominal cavity of cervids. Setariae are commonly believed to be non-pathogenic in their natural hosts but severe disease outbreaks with associated peritonitis and perihepatitis caused by *S. tundra* have been reported [18]. In Scandinavian countries, reindeer is the main vertebrate host [19] whereas in Central Europe only roe and red deer have been found parasitized so far [20-22]. Human infections have not been described.

Dirofilariae are transmitted by culicid mosquitoes (Diptera, Culicidae) of various species, such as *Cx. pipiens*, *Anopheles maculipennis* s.l. and *Aedes albopictus,* which are probably the most important vectors in the Mediterranean [23-25]. Some of these, such as *Cx. pipiens* and *An. maculipennis* s.l., are widely distributed over Europe while others, such as *Ae. albopictus*, are expanding northwards from established distribution areas in the Mediterranean [26]. The main vectors of *S. tundra* are supposed to belong to the genus *Aedes*[27].

Within a German national mosquito monitoring programme launched in 2011, mosquito samples were screened for various pathogens such as viruses and filarial nematodes. We describe here the finding of at least four filarial nematode species in mosquitoes collected in Germany, including the first detection of *D. immitis* in Germany.

## Methods

Adult mosquitoes were collected at numerous sites all over Germany between 2011 and 2013 using BG sentinel traps (Biogents, Germany) equipped with BG Lure™ and CO_2_ as attractants, or by hand. The mosquitoes were caught by trained non-specialists who kept them frozen until further processing. Upon transportation to the laboratory, the mosquitoes were identified morphologically [28,29] or genetically, following RNA/DNA extraction as described below. Specifically, Maculipennis Group species (*An. maculipennis* s.l.) were identified by species-specific PCR [30], whereas mosquitoes neither identifiable morphologically nor by PCR were subjected to COI barcoding [31].

A total of approximately 17,000 female mosquitoes belonging to six genera (*Aedes*, *Anopheles*, *Coquillettidia*, *Culex*, *Culiseta* and *Ochlerotatus*) were pooled by species, collection site and date with up to 25 specimens per pool. Mosquitoes identified by COI barcoding or species-specific PCR represented pools consisting of one specimen only. Each pool was homogenized in the presence of stainless steel beads (diameter 3 mm) in a maximum of 750 μl minimum essential medium (MEM) containing 10 μg/ml gentamicin, 0.25 μg/ml amphotericin B, 100 U/ml penicillin and 100 μg/ml streptomycin by a TissueLyserII (Qiagen, Germany) for 3 min at 30 Hz. The homogenate was centrifuged for 1 min at 14,000 g, and the supernatant was used for simultaneous RNA/DNA extraction by means of the NucleoSpin 96 Virus Core Kit (Macherey-Nagel, Germany) according to the user manual.

For screening the mosquito pools for filarial nematodes (Filarioidea), a filarioid-specific real-time PCR assay was developed targeting a 90 bp fragment of the mitochondrial 16S rRNA gene with the newly designed primers PanFilaF (5’-TGTGCTGCGCTACATCGATG-3’) and PanFilaR (5’-AAACCGCTCTGTCTCACGAC-3’). The primers were constructed after alignment of partial and complete mitochondrial genome sequences of nine parasitic filarial nematode species (*D. immitis, D. repens, S. tundra, Setaria digitata, Brugia malayi, Wuchereria bancrofti, Onchocerca flexuosa, Onchocerca volvulus*), which are epidemiologically important in human and animal health, and, additionally, are appropriately represented in GenBank. Sequences were analysed with BioEdit Sequence Alignment Editor [32], and conserved DNA regions were identified for primer design taking into account standard rules of designing primers for real-time PCR assays [33]. Specificity of the primers was confirmed on *D. immitis*, *D. repens*, *O. volvulus* and *W. bancrofti* DNA. A more in-depth testing was not considered necessary as the PCR was meant for sample screening only, thus possibly allowing false negative but not false positive results. The real-time PCR was performed using the CFX96 Touch™ Real-Time PCR Detection System (BioRad, Germany) and ResoLight non-specific detection chemistry, followed by high-resolution melting-analysis. The reaction mixture (25 μl) contained 1 μl ResoLight dye (Roche Diagnostics, Germany), 10 μl of 2× QuantiTect Multiplex PCR Master Mix (Qiagen, Germany), 0.4 μM forward and reverse primer each, and 5 μl of extracted DNA. The thermoprofile consisted of an initial denaturation step at 95°C for 15 min, 35 cycles of 95°C for 45 sec, 58°C for 30 sec and 72°C for 45 sec, and a final extension step at 72°C for 5 min. All amplifications and detections were carried out in Multiplate™ Low-Profile 96-Well PCR Plates with optical Microseal 'B' Film (BioRad). After each annealing cycle, accumulation of PCR products was detected by monitoring the increase in fluorescence of double-stranded DNA-binding ResoLight at 518 nm. After the PCR, a dissociation curve was constructed for steps of 5°C in the range from 60°C to 95°C. All data were analyzed using the BioRad CFX-Manager software.

Samples yielding a signal in the real-time PCR were processed by a second conventional PCR amplifying about 650 bp of the filarioid COI gene [34]. After agarose gel electrophoresis, PCR products were excised from the gels and recovered by the QIAquick Gel Extraction Kit (Qiagen, Germany). They were cycled bidirectionally using the BigDye Terminator v1.1 Cycle Sequencing Kit (Applied Biosystems, Germany), and sequencing products were purified by SigmaSpin Sequencing Reaction Clean-Up Columns (Sigma Aldrich, Germany) before loading onto a 3130 Genetic Analyser (Applied Biosystems). For species identification, consensus sequences of positive samples were compared with sequences available in GenBank.

## Results

**Figure 1:**
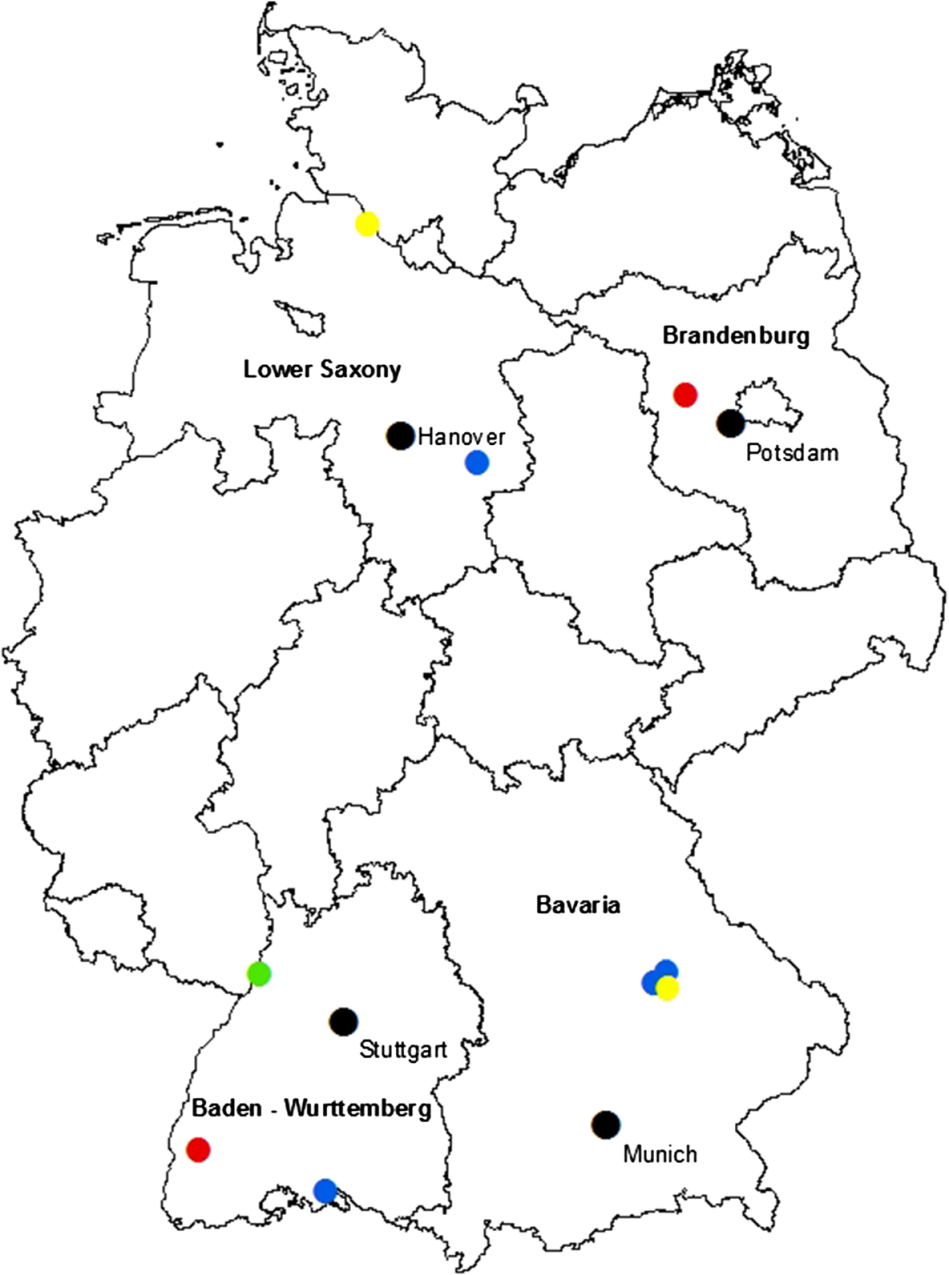
**Geographic origin of the mosquitoes tested positive (red dots: *D. immitis*, green dot: *D. repens*, blue dots: *S. tundra*, yellow dots: filariae of unknown species).**

16,878 female mosquitoes representing 16 species or species complexes in six genera were processed, with *Cx. pipiens/torrentium* (73%), *Anopheles plumbeus* (11%) and *Aedes vexans* (8%) being the most frequent mosquito taxa examined (Table 1). In total, 955 pools were screened using the pan-filarioid real-time PCR. Nine pools (0.94%) testing positive were confirmed to contain filarioid DNA by the COI PCR assay. Of the sequences obtained, two showed 100% and 99% identity to *D. immitis* (Table 2) while presenting two nucleotide differences in direct comparison. One sequence displayed 99% identity to *D. repens* and four sequences exhibited 99% identity to *S. tundra*, with the *S. tundra* sequences being variable among each other at six positions. Two positive samples, displaying 91% homology in direct comparison, could not be assigned to a species due to insufficient identities to GenBank entries (89 and 92% maximum; Table 2). The *D. immitis*-positive pools as well as those with unknown filarioid DNA sequences were composed of *Cx. pipiens/torrentium* specimens, the *D. repens*-positive pool was equivalent to a single *An. daciae* female, and the *S. tundra*-positive pools contained *Ae. vexans* mosquitoes (Table 2). All filariae-carrying culicids had been collected between mid-June and early September in 2011, 2012 and 2013 in four German federal states (Table 2, Figure 1).

**Table 1:** Mosquito species and pools examined

**Species**	**No. of mosquitoes tested (%)**	**No. of pools tested/positive**
*Culex pipiens/torrentium*	12,292 (72.83)	554/4
*Anopheles plumbeus*	1,843 (10.9)	93/0
*Aedes vexans*	1,356 (8.03)	111/4
*Aedes cinereus/geminus*	451 (2.67)	22/0
*Anopheles maculipennis* s.l.*	336 (2)	99/1
*Culiseta annulata*	253 (1.5)	39/0
*Coquillettidia richardii*	132 (0.8)	10/0
*Ochlerotatus diantaeus*	73 (0.43)	5/0
*Ochlerotatus cantans/annulipes*	58 (0.34)	7/0
*Anopheles claviger*	50 (0.30)	6/0
*Ochlerotatus leucomelas*	12 (0.07)	1/0
*Ochlerotatus detritus*	10 (0.06)	1/0
*Ochlerotatus excrucians*	6 (0.04)	1/0
*Ochlerotatus caspius*	3 (0.02)	3/0
*Ochlerotatus sticticus*	2 (0.01)	2/0
*Culiseta alaskaensis*	1 (0.01)	1/0
Total	16,878	955/9

*including *An. daciae*.

**Table 2:** Collection details of filarioid-positive mosquito pools (BW: federal state of Baden-Wurttemberg, BB: Brandenburg, LS: Lower Saxony, BV: Bavaria)

**Filarial species**	**GenBank accession no.**	Max.% identity to GenBank entry (accession no.)*	**Collection site**	**Collection date**	**Mosquito species**	**Pool size (no. mosquitoes)**
*D. immitis*	KF692100	100% (e.g. EU159111)	Freiburg (BW)	07-13-2012	*Cx. pipiens/torrentium*	25
*D. immitis*	KF692101	99% (e.g. EU163945)	Buschow (BB)	08-23-2012	*Cx. pipiens/torrentium*	5
*D. repens*	KF692102	99% (e.g. DQ358814)	Eggenstein-Leopoldshafen (BW)	08-03-2012	*An. daciae*	1
*S. tundra*	KF692103	99% (e.g. DQ097309)	Braunschweig (LS)	08-22-2012	*Ae. vexans*	20
*S. tundra*	KF692104	99% (e.g. DQ097309)	Radolfzell (BW)	08-02-2012	*Ae. vexans*	21
*S. tundra*	KF692105	99% (e.g. DQ097309)	Regensburg (BV)	09-02-2011	*Ae. vexans*	10
*S. tundra*	KF692106	99% (e.g. DQ097309)	Regensburg (BV)	07-07-2013	*Ae. vexans*	25
Filarioidea	-	89% (HQ186250)	Regensburg (BV)	06-17-2011	*Cx. pipiens/torrentium*	9
Filarioidea	-	92% (JX870433)	Drochtersen (LS)	08-02-2012	*Cx. pipiens/torrentium*	25

*as of 07 November 2013.

The COI DNA sequences of the identified worms found in the mosquitoes have been deposited in GenBank under accession numbers KF692100-KF692106.

## Discussion

As dirofilariosis is a vector-borne disease, its epidemiology is highly susceptible to climatic and environmental conditions. In the recent past, it has become an emerging problem in numerous countries of the world, including many European ones [1,7]. The two causative agents in Europe, *D. immitis* and *D. repens*, have been found outside their traditional distribution ranges in the Mediterranean with increasing frequency. In particular, in Central and eastern European states, such as Austria, Hungary, Slovakia and Poland, autochthonous cases have been diagnosed in dogs and humans [11,16,35-38].

With *D. immitis* and *D. repens*, two mosquito-borne zoonotic filarial nematode species endemic to southern Europe were detected in mosquitoes collected in Germany. Hence, this is the first report from Germany of *D. immitis* apparently acquired locally.

Notably, the various filarial species were demonstrated in their putative vectors which included *An. daciae*, a recently recognized member of the Maculipennis Group [39], a complex of several closely related isomorphic *Anopheles* species, in addition to two previously described potential vectors of the filariae, *Ae. vexans* and *Cx. pipiens* (due to the pooling of the mosquitoes, a molecular differentiation between the morphologically indistinguishable females of *Cx. pipiens* and *Cx. torrentium* was not carried out in this study). The detection of the filariae in these mosquito species is not surprising as Cancrini *et al.*[23,24] found *D. repens* in *Cx. pipiens* and Maculipennis Group specimens in Italy while Bocková *et al*. [40] only recently reported *D. repens* in a pool of *Ae. vexans* mosquitoes from Slovakia. *Dirofilaria immitis* has been described from *Cx. pipiens* in Spain [41], and from *Ae. vexans* and *Cx. pipiens* in Turkey [42].

The finding of two unknown filarial species in *Cx. pipiens/torrentium* mosquito pools suggests avian bloodmeal sources of the mosquitoes due to the feeding preferences within this group of culicids, and therefore avian nematode species, one of them possibly being *Cardiofilaria pavlovskyi*, as discussed by Cjaka *et al*. [43].

The route through which the dirofilariae found their way to Germany or the sources of filarial ingestion by the mosquitoes, respectively, must remain speculative. The *D. immitis*-positive mosquito/es from Baden-Wurttemberg was/were collected at the same site where *Ae. albopictus* had repeatedly been trapped previously [26]. This site is characterized by its close proximity to a railway transshipment station where cargo from trucks coming in from southern Europe is transferred to trains. Hence, it is conceivable that, as with *Ae. albopictus*, the filarioid-positive *Cx. pipiens*/*torrentium* mosquito/es was/were introduced from southern Europe by vehicle transport. By contrast, the finding of *D. immitis* in a pool of mosquitoes from Brandenburg must be attributed most probably to a local uptake by the feeding mosquito/es. A possible source might have been a dog imported from, or with a travel history to, southern Europe. The detection of *D. repens* in *An. daciae* is noteworthy not only because nothing is known about the vector potential of this mosquito species but also because it was collected in the same area where *D. repens* had been isolated from dogs in 2007 [12]. Possibly, a local transmission cycle has established in that area.

The third mosquito-borne filarial nematode described, *S. tundra*, seems to be more common in Germany than generally assumed, as it had been detected microscopically or by PCR on several occasions in the past [22,43]. Detailed studies regarding its abundance, distribution and even pathology in areas south of Scandinavia, however, are lacking.

As surprising as the dirofilarial findings are, Genchi and colleagues [5,44] considered both Baden-Wurttemberg and Brandenburg as climatically suitable for dirofilarial development in mosquitoes and assigned to these regions a risk of stable endemicity.

Simón *et al.*[1] calculated a transmission period of 3–4 months in Central Europe for both *D. repens* and *D. immitis*, taking into account the extrinsic incubation periods of the worms. At a mean temperature of 18°C, for example, these will last about 29 days, at 22°C still 16–20 days. *Setaria tundra* needs an average of 14–16 days at 21°C to reach the metacyclic infectious L3 stage in the mosquito [45]. Considering the lifespan of a mosquito of a few weeks at its best, these long developmental periods may presently limit the rate of dispersal of the filariae. However, as hot summer periods are predicted to become more frequent and longer as a result of climate change, mosquito-borne filarioses will probably become a growing problem to veterinary and human health in Central and eastern Europe in the future.

## Conclusion

With progressing globalization and climate change, the risk of the introduction of zoonotic *D. immitis* and *D. repens* from endemic areas in southern Europe to previously infection-free areas in northern Europe and their subsequent establishment increases. Vector-competent mosquitoes are probably already present there, and the climatic conditions are regionally and seasonally adequate for the filariae to finish their development in infected mosquitoes. The possibility of dirofilarial infections in dogs and other carnivores as well as in humans should therefore be considered with regard to differential diagnosis in unclear cases of appropriate symptomatology.

No information exists on a possible spread and an increase in prevalence of *S. tundra* in Central Europe as respective studies are missing. Although this worm does not appear to significantly affect the health of indigenous cervids at present, further research on the epidemiology of setariosis in Central Europe is desirable.

## Abbreviations

COI: Cytochrome c oxidase subunit I.

## Competing interests

The authors declare that they have no competing interests.

## Authors’ contributions

MK, HK and DW designed the study, collected and identified the mosquitoes and contributed to the data analysis and writing of the manuscript. MK established the detection techniques and screened the mosquitoes in the laboratory. HK and DW supervised the field and laboratory work. RS organized mosquito trapping in BB and added to preparing the manuscript. All authors read and approved the final version of the manuscript.
